# Combined pyrotinib and fulvestrant for hormone receptor‐positive and HER2‐positive metastatic breast cancer: A multicenter, single‐arm, phase II trial

**DOI:** 10.1002/mco2.70031

**Published:** 2024-12-20

**Authors:** Jianli Zhao, Yunfang Yu, Wei Ren, Linxiaoxiao Ding, Yongjian Chen, Peng Yuan, Jian Yue, Yaping Yang, Guorong Zou, Tao Chen, Jie Chai, Li Zhang, Wenjing Wu, Yinduo Zeng, Xiujuan Gui, Yangyang Cai, Simin Luo, Zhongyu Yuan, Kang Zhang, Herui Yao, Ying Wang

**Affiliations:** ^1^ Guangdong Provincial Key Laboratory of Malignant Tumor Epigenetics and Gene Regulation Department of Medical Oncology Breast Tumor Centre Phase I Clinical Trial Centre, Clinical Research Design Division, Clinical Research Center Sun Yat‐sen Memorial Hospital Sun Yat‐sen University Guangzhou Guangdong China; ^2^ Faculty of Medicine Macau University of Science and Technology Taipa Macao PR China; ^3^ Dermatology and Venereology Division Department of Medicine Solna Center for Molecular Medicine Karolinska Institute Stockholm Stockholm Sweden; ^4^ Department of VIP Medical Services National Cancer Center/National Clinical Research Center for Cancer/Cancer Hospital Chinese Academy of Medical Sciences and Peking Union Medical College Beijing China; ^5^ Department of Medical Oncology the Affiliated Panyu Central Hospital of Guangzhou Medical University Guangzhou Guangdong China; ^6^ Department of Medical Oncology Sun Yat‐Sen University Cancer Center the State Key Laboratory of Oncology in South China Collaborative Innovation Center for Cancer Medicine Guangzhou Guangdong China; ^7^ Guangzhou National Laboratory Guangzhou Guangdong China; ^8^ Advanced Institute for Eye Health and Diseases Wenzhou Medical University Wenzhou China

## Abstract

This multicenter, single‐arm, phase II clinical trial (NCT04034589) evaluated the efficacy and safety of pyrotinib combined with fulvestrant in patients with HR‐positive/HER2‐positive metastatic breast cancer who had experienced trastuzumab treatment failure. A total of 46 patients were enrolled, receiving pyrotinib orally once daily and fulvestrant intramuscularly on days 1 and 15 of cycle 1, followed by monthly doses on day 1. The primary endpoint was progression‐free survival (PFS), while secondary endpoints included overall survival (OS), objective response rate (ORR), disease control rate (DCR), and safety. The median PFS was 18.2 months (95% CI, 11.9–31.1) overall, 19.5 months (95% CI, 10.6–NA) for those receiving the combination as first‐line therapy, and 18.4 months (95% CI, 16.7–NA) for patients with brain metastases. Median OS was not reached, with a 3‐year OS rate of 75.2% (95% CI, 62.8–90.2%). The ORR was 32.5%, and the DCR was 97.5%. Responses were observed in patients with low tumor mutation burden and *ZNF217* mutation. Importantly, no grade 4 or higher treatment‐related adverse events or deaths were reported, indicating a favorable safety profile. In conclusion, the combination of pyrotinib and fulvestrant demonstrated promising antitumor activity and acceptable safety in HR‐positive/HER2‐positive metastatic breast cancer patients.

## Graphical abstract

This study suggests that pyrotinib plus fulvestrant might be an efficacious and well‐tolerated option in HR‐positive/HER2‐positive metastatic breast cancer patients who have shown resistance to trastuzumab. This regimen could be a potential first‐line treatment and may be considered the preferred option for patients with brain metastases, secondary resistance to trastuzumab, and those who have received adjuvant endocrine therapy. Additionally, responses were observed in patients with low tumor mutation burden and *ZNF217* mutation

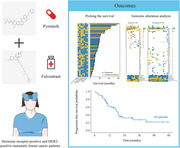

## 1 INTRODUCTION

Human epidermal growth factor receptor 2 (HER2) gene amplification and overexpression occur in roughly 20% of breast cancer cases, about half of which are hormone receptor (HR)‐positive.^1^ Patients diagnosed with HR‐positive/HER2‐positive subtype tend to exhibit slow proliferation and poor sensitivity to chemotherapy.^2^ HR status is often not emphasized in treatment guidelines for HER2‐positive metastatic breast cancer. Current recommendations endorse anti‐HER2 monoclonal antibody therapies combined with taxane chemotherapy as the standard first‐line systemic treatment for these patients.^3^, ^4^, ^5^ However, the role of endocrine therapy in the treatment of HR‐positive/HER2‐positive breast cancer and the selection of targeted drugs after the failure of trastuzumab treatment have emerged as new hurdles.

Previous clinical trials have initially confirmed the efficacy of endocrine therapy combined with HER2‐targeted therapy, such as the MUKDEN 01 study in a neoadjuvant setting,^6^ and TAnDEM study,^7^ monarchE study,^8^ EGF30008 study,^9^ and PLEHERM study^10^ for metastatic breast cancer. Especially in the randomized controlled SYSUCC‐002 trial, 392 patients were assigned at random to receive either trastuzumab with endocrine therapy or trastuzumab combined with chemotherapy as a first‐line treatment for HER2‐positive metastatic breast cancer.^11^ With a median follow‐up of 30.2 months, the median progression‐free survival (PFS) was 19.2 months in the endocrine therapy group compared with 14.8 months in the chemotherapy group (hazard ratio 0.88, *p* < 0.0001). However, the combination of endocrine therapy with HER2‐targeted therapy is still typically considered for patients who are unable to tolerate chemotherapy, and the treatment choice after trastuzumab resistance is currently a focal point of research in this field.

Earlier research suggests that targeting HER2 in isolation can activate HR due to complex interactions and crosstalk between the HR and HER2 signaling pathways. This mechanism may enable tumor cells to evade treatment, resulting in resistance to anti‐HER2 therapies.^12^, ^13^ Targeting HER2 represents a crucial strategy for overcoming estrogen receptor (ER) resistance. Pyrotinib is an irreversible small‐molecule tyrosine kinase inhibitor that targets multiple HER family receptors, including epidermal growth factor receptor (EGFR), HER2, and HER4.^14^ Informed by the results of the PHILA and PHEDRA phase 3 studies, pyrotinib, combined with the anti‐HER2 monoclonal antibody trastuzumab and docetaxel, has gained approval in China for treating HER2‐positive breast cancer in both first‐line and neoadjuvant settings.^1^, ^15^

The selective estrogen receptor degrader fulvestrant was demonstrated to have soupier efficacy than third‐generation aromatase inhibitors, such as anastrozole, and exhibits a synergistic effect with anti‐HER2 medications.^16^, ^17^ Currently, data on the clinical evaluation of combination therapy with pyrotinib and fulvestrant for HR‐positive/HER2‐positive metastatic breast cancer remain limited. In this context, we conducted a prospective, multicenter, single‐arm phase II study to evaluate the efficacy and safety of pyrotinib combined with fulvestrant in patients with HR‐positive/HER2‐positive metastatic breast cancer.

## 2 RESULTS

### 2.1 Study participants

**FIGURE 1:**
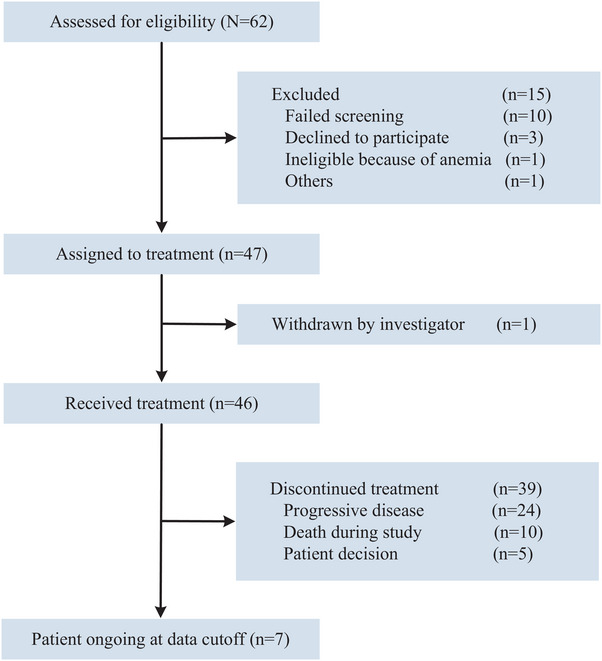
Study flow diagram.

A total of 62 patients were screened between March 2019 and March 2022, 46 patients were finally enrolled from 5 study centers and all received study treatment. The disposition of the enrolled patients is presented in the flowchart (Figure 1). The median age was 55.0 years (interquartile range [IQR], 46.0–59.0). As of the data cutoff on August 15, 2024, the median duration of follow‐up was 32.0 months (IQR, 24.0–43.0). Treatment was discontinued in 39 patients (84.8%) due to disease progression (24, 52.2%), death (10, 21.7%), and patient decision (5, 10.9%).

The patient characteristics are summarized in Table 1. Of the participants enrolled, 43% had visceral metastasis, 13% had brain metastasis, and 59% and 41% received study treatment as first‐ or second‐line therapy, respectively, and 59% had an IHC3+ HER2 status. A total of 24 patients (52%) received adjuvant endocrine therapy and 13 patients (28%) underwent radiotherapy. Among eligible patients, 29 (63%) and 23 (50%) patients received trastuzumab as adjuvant or first‐line therapy, respectively. Additionally, De novo resistance to trastuzumab was observed in 21 patients (46%), while 25 patients (54%) had secondary resistance. Thirty specimens were collected from biopsy or surgical samples prior to the initiation of study treatment.

**TABLE 1:** Baseline characteristics.

Variable, *N* (%)	*N* = 46
Median follow‐up (months; median, IQR)	32 (24, 43)
Age (months; median, IQR)	55 (46, 59)
<40	7 (15%)
≥40	39 (85%)
HER2 status	
2+	19 (41%)
3+	27 (59%)
ER status	
<50	14 (30%)
≥50	32 (70%)
PR status	
<50	36 (78%)
≥50	10 (22%)
Lines of treatment	
First‐line	27 (59%)
Second‐line	19 (41%)
Visceral metastasis	
No	26 (57%)
Yes	20 (43%)
Brain metastases	
No	39 (87%)
Yes	6 (13%)
Bone metastases only	
No	38 (82.6%)
Yes	8 (17.4%)
Radiotherapy	
No	33 (72%)
Yes	13 (28%)
Adjuvant endocrine therapy	
No	22 (48%)
Yes	24 (52%)
Adjuvant trastuzumab therapy	
No	17 (37%)
Yes	29 (63%)
Trastuzumab (1 line)	
No	23 (50%)
Yes	23 (50%)
Trastuzumab secondary resistance	
No	21 (46%)
Yes	25 (54%)

Abbreviations: ER, estrogen receptor; HER2, human epidermal growth factor receptor 2; IQR, interquartile range; PR, progesterone receptor.

### 2.2 Efficacy

**FIGURE 2:**
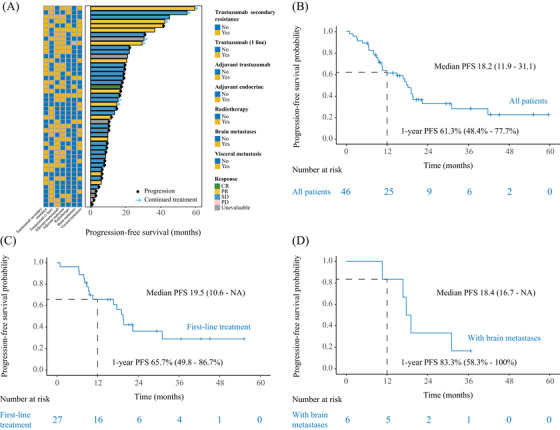
Estimates of progression‐free survival. (A) The progression‐free survival distributions among enrolled patients. (B) Progression‐free survival in total population. (C) Progression‐free survival among patients who received pyrotinib plus fulvestrant as first‐line treatment. (D) Progression‐free survival among patients with brain metastases. We calculated *p*‐values using the unadjusted log‐rank test and hazard ratios using univariate Cox regression analysis. CI, confidence interval; HR, hazard ratio; PFS, progression‐free survival.

After a median follow‐up of 32.0 months, with 29 patients experiencing a PFS event, the median PFS was 18.2 months (95% confidence interval [CI], 11.9–31.1) and 1‐year PFS rate was 61.3% (95% CI, 48.4–77.7%) (Figure 2A,B). Furthermore, the median PFS achieved 19.5 months (95% CI, 10.6–NA; Figure 2C) and 18.4 months (95% CI, 16.7–NA; Figure 2D) among patients treated with pyrotinib plus fulvestrant as first‐line treatment and patients with brain metastases, respectively. Patients with bone metastases only showed prolonged PFS with study treatment (HR, 0.18 [95% CI, 0.04–0.79]; *p* = 0.011; Figure S1). The PFS distributions and treatment effects were similar among patients who experienced radiotherapy, adjuvant endocrine therapy, or adjuvant trastuzumab therapy (Table S1).

The response data are shown in Table 2. The secondary endpoint of the confirmed objective response rate (ORR) was 32.5% with 13 of 40 patients, including one complete response and 12 partial responses. The confirmed ORR reached 41.7% for patients with HER2 3+, 57.1% for those with progesterone receptor (PR) expression over 50%, 44.4% for patients with visceral metastasis, 40.0% for those with brain metastases, 53.3% for patients who had not received adjuvant trastuzumab therapy, and 40.0% for those experiencing secondary resistance to trastuzumab (Figure S2). The confirmed disease control rate (DCR) was 97.5%.

**TABLE 2:** Confirmed tumor response.

Tumor response, *N* (%)	*N* = 40
Objective response rate	13 (32.5%)
Disease control rate	39 (97.5%)
Complete response	1 (2.5%)
Partial response	12 (30.0%)
Stable disease	26 (65.0%)
Progressive disease	1 (2.5%)
Unevaluable	6 (13.0%)

*Note*: Unevaluable indicates bone metastases only were not measurable.

**FIGURE 3:**
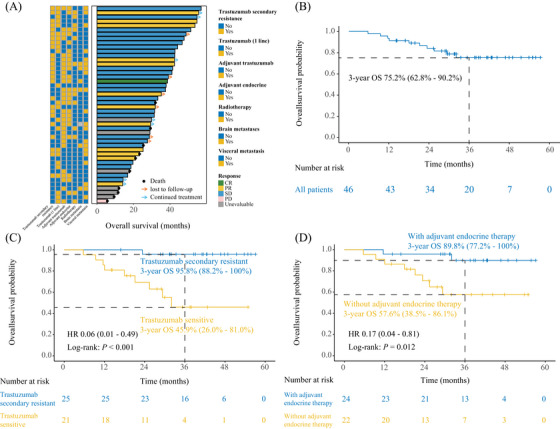
Estimates of overall survival. (A) The overall survival distributions among enrolled patients. (B) Overall survival in total population. (C) Kaplan–Meier curves for overall survival by the subcategory of secondary trastuzumab resistance. (D) Kaplan–Meier curves for overall survival by the subcategory of adjuvant endocrine therapy. We calculated *p*‐values using the unadjusted log‐rank test and hazard ratios using univariate Cox regression analysis. CI, confidence interval; HR, hazard ratio; OS, overall survival.

The median overall survival (OS) for the total population has not been reached. Ten patients succumbed to tumor progression, yielding a 3‐year OS rate of 75.2% (95% CI, 62.8–90.2%; Figure 3A,B). Among patients with trastuzumab secondary resistant disease, the median OS was 32.0 months, which showed better OS with study treatment than patients with trastuzumab‐sensitive disease (HR, 0.06 [95% CI, 0.01–0.49]; *p* < 0.001; Figure 3C). Patients who received adjuvant endocrine therapy seemed to have longer OS than patients without adjuvant endocrine therapy (HR, 0.17 [95% CI, 0.04–0.81]; *p* = 0.012; Figure 3D). Table S2 shows the OS distributions among subgroups of patients.

### 2.3 Safety

The safety analysis included all enrolled patients (*n* = 46). Table 3 summarizes treatment‐related adverse events. Grade 3 or higher events occurred in 43.5% (20/46) of patients. No patients discontinued treatment or required dose reductions due to adverse reactions. Diarrhea was the most frequent grade 3 or higher event, affecting 26.1% of patients. There were no grade 4 events or treatment‐related deaths.

**TABLE 3:** Treatment‐related adverse events.

Adverse events, *N* (%)	Grade 1 (*N* = 39)	Grade 2 (*N* = 18)	Grade 3 (*N* = 18)
Hematological			
Neutropenia	3 (6.52%)	0 (0.0%)	0 (0.0%)
Lymphocytopenia	7 (15.22%)	0 (0.0%)	2 (4.35%)
Leukopenia	4 (8.70%)	2 (4.35%)	0 (0.0%)
Anemia	4 (8.70%)	7 (15.22%)	0 (0.0%)
Metabolism and nutrition disorders			
Hyponatremia	4 (8.70%)	0 (0.0%)	0 (0.0%)
Hypokalemia	4 (8.70%)	0 (0.0%)	1 (2.17%)
Decreased appetite	3 (6.52%)	0 (0.0%)	0 (0.0%)
Gastrointestinal			
Constipation	3 (6.52%)	0 (0.0%)	0 (0.0%)
Vomiting	6 (13.04%)	0 (0.0%)	1 (2.17%)
Nausea	4 (8.70%)	2 (4.35%)	0 (0.0%)
Diarrhea	16 (34.78%)	11 (23.91%)	12 (26.09%)
Abdominal pain	6 (13.04%)	0 (0.0%)	0 (0.0%)
Abdominal distension	0 (0.0%)	0 (0.0%)	1 (2.17%)
Other			
Oral mucositis	4 (8.70%)	0 (0.0%)	0 (0.0%)
Cough	2 (4.35%)	0 (0.0%)	0 (0.0%)
Hand‐foot syndrome	9 (19.57%)	2 (4.35%)	0 (0.0%)
Hot flashes	2 (4.35%)	0 (0.0%)	0 (0.0%)
Fatigue	8 (17.39%)	0 (0.0%)	0 (0.0%)
Rash	6 (13.04%)	1 (2.17%)	1 (2.17%)
Skin ulcer	0 (0.0%)	0 (0.0%)	1 (2.17%)
Elevated creatinine	8 (17.39%)	2 (4.35%)	0 (0.0%)
Elevated ALT	1 (2.17%)	1 (2.17%)	0 (0.0%)
Elevated AST	5 (10.87%)	0 (0.0%)	1 (2.17%)
Dizziness	3 (6.52%)	0 (0.0%)	0 (0.0%)
Headache	3 (6.52%)	0 (0.0%)	0 (0.0%)
Myalgia	1 (2.17%)	0 (0.0%)	0 (0.0%)
Arthralgia	4 (8.70%)	0 (0.0%)	0 (0.0%)
Bone pain	1 (2.17%)	0 (0.0%)	0 (0.0%)
Other	10 (21.74%)	0 (0.0%)	0 (0.0%)

Abbreviations: ALT, Alanine aminotransferase; AST, Aspartate aminotransferase.

### 2.4 Exploration of biomarkers

**FIGURE 4:**
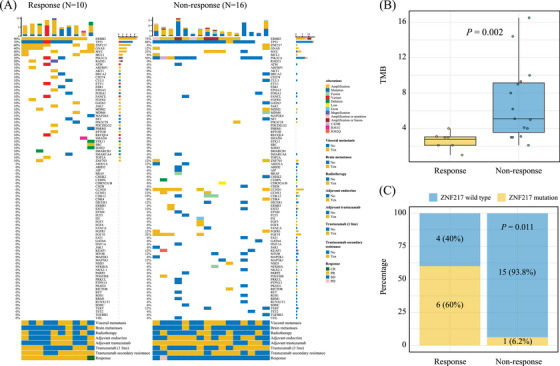
Individual gene mutations associated with response. (A) The oncoprint of individual gene mutations among response and nonresponse patients. (B) Response patients emerged with low tumor mutation burden. (C) Mutations in *ZNF217* were associated with responsiveness.

To explore the correlation of biomarkers with the efficacy and safety of pyrotinib and fulvestrant, tumor tissue samples were collected for the detection of gene mutations (Figure S3). Mutations in *PIK3CA* (response group, 2 of 10 [20%]; nonresponse group, 9 of 16 [56%]) and *TP53* (response group, 7 of 10 [70%]; nonresponse group, 14 of 16 [88%]) were common and associated with increased unresponsiveness but did not reach statistical significance (Figure 4A). Compared with response patients, nonresponse patients emerged with higher tumor mutation burden (TMB) (*p* = 0.002, Figure 4B). We identified that mutations in *ZNF217* were associated with responsiveness (response group, 6 of 10 [60%]; nonresponse group, 1 of 16 [6%]; *p* = 0.011; Figure 4C).

## 3 DISCUSSION

To the best of our knowledge, this trial represents the first assessment of the combination therapy involving pyrotinib and fulvestrant in patients with HR‐positive/HER2‐positive breast cancer who have experienced progression after trastuzumab treatment. The findings indicate that this combination results in a median time to progression of 18.2 months and a 3‐year OS rate of 75.2%, accompanied by a favorable safety profile. Importantly, for those receiving this combination as first‐line therapy, the median PFS was recorded at 19.5 months, while it was 18.4 months for patients with brain metastases. These outcomes imply that combining pyrotinib with fulvestrant may be an effective approach for individuals with HR‐positive/HER2‐positive breast cancer following disease progression on trastuzumab. Furthermore, this combination has shown efficacy in patients exhibiting low TMB and possessing a *ZNF217* mutation.

Preclinical studies show that in mouse xenograft models of ER‐positive/HER2‐positive breast cancer, treatment with lapatinib alone or combined with trastuzumab leads to heightened ER activity, contributing to anti‐HER2 resistance.^12^ We demonstrated for the first time in a clinical study that the combination of pyrotinib and fulvestrant is effective. Although the DCR was high in this study, the ORR was relatively low, which is attributed to the less aggressive biological behavior of HR‐positive/HER2‐positive breast cancer. This suggests that chemotherapy is not suitable, combining pyrotinib with fulvestrant offers a promising benefit.

In this study, the median PFS achieved 19.5 months among patients treated with pyrotinib plus fulvestrant as first‐line treatment, which is statistically comparable to the 18.7 months of PFS observed with the THP standard treatment strategy in the Cleopatra study.^18^ This result preliminarily suggests that the combination of fulvestrant and pyrotinib could be a potential first‐line treatment option for HR‐positive/HER2‐positive metastatic breast cancer patients.

Antibody–drug conjugates are continuously emerging and evolving.^19^ Trastuzumab deruxtecan (T‐Dxd) has been the standard treatment following trastuzumab treatment failure with a PFS of 28.8 months.^20^ However, long‐term administration of T‐Dxd poses a substantial challenge due to its significant adverse reactions. Developing a step‐down therapy approach, including selecting a low‐toxicity sequential regimen has become a pressing clinical issue. The PFS of 18 months in patients treated with fulvestrant plus pyrotinib as second‐line therapy observed in this study, suggests that the combination is a competitive strategy for HR‐positive/HER2‐positive metastatic breast cancer. It may also serve as an alternative for patients with high tumor burden who have already benefited from T‐Dxd.

Brain metastases are a significant concern in HER2‐positive breast cancer with poor prognosis, due to the limited efficacy of antitumor therapies in overcoming the blood–brain barrier.^21^ The LANDSCAPE study demonstrated that the combination of lapatinib and capecitabine achieved an ORR of 57.1% and a median time to tumor progression of 5.5 months in patients with HER2‐positive newly active brain metastasis.^22^ Similarly, the PERMEATE study showed that pyrrotinib combined with capecitabine resulted in a central nervous system ORR of 74.6% and a median PFS of 11.3 months for newly active brain metastases.^23^ The results of this study indicate that fulvestrant combined with pyrotinib achieved a PFS of 18.4 months and an intracranial ORR of 40% in patients with brain metastases, suggesting that this combination is also a promising disease control strategy for these patients.

Overall, the adverse events in this study were manageable and consistent with those reported in previous pyrotinib trials. Diarrhea was the most frequent adverse event, with a grade ≥3 incidence of 26%—lower than the 31% observed in the PHOEBE trial (pyrotinib plus capecitabine)^24^ and 46% in the PHILA trial (pyrotinib combined with trastuzumab and docetaxel).^1^ This side effect was effectively controlled through dose adjustments and antidiarrheal treatments, such as montmorillonite powder or loperamide, without leading to treatment discontinuation. Among patients treated with pyrotinib and fulvestrant, 43% experienced grade 3 adverse events, but no serious adverse events were observed. These results indicate that the regimen is well‐tolerated and could be a viable alternative for patients seeking to avoid the side effects of traditional chemotherapy.

Combining pyrotinib with fulvestrant has proven beneficial to some specific patients, the challenge lies in exploring biomarkers to identify which patients will benefit most from the therapy strategy. A prior study revealed that *PIK3CA* mutations correlate with reduced pCR rates in HER2‐positive breast cancer patients receiving neoadjuvant anti‐HER2 therapy.^25^ In this study, mutations in *PIK3CA* also showed an association with increased unresponsiveness but did not reach statistical significance due to the small sample size. Additionally, a study identified *ZNF217* with increased mutation frequency after anti‐EGFR therapy in metastatic colorectal cancer patients,^26^ while a mutation in *ZNF217* and low TMB in this study were found to be significantly associated with treatment response. Long noncoding RNAs, multimodal biomedical, immune cells, and cytokines have potential as predictive markers^27^, ^28^, ^29^, ^30^; future studies based on single‐cell and RNA sequencing could further elucidate therapeutic efficacy and underlying mechanisms.^31^

The significance of this study lies in confirming the synergistic effect of fulvestrant combined with pyrotinib and providing a low‐toxicity, high‐efficiency treatment option without chemotherapy for HR‐positive/HER2‐positive metastatic breast cancer patients. In a future Phase III study, we plan to compare first‐line treatment with fulvestrant plus pyrotinib (with the potential addition of CDK4/6 inhibitors) against the standard first‐line THP regimen. To accelerate the process, we will leverage advanced technologies to rapidly screen potential trial participants, optimize trial design, monitor and analyze trial data in real‐time, and develop biomarkers through the integration of artificial intelligence with multimodal data.^32^, ^33^, ^34^

However, this study has several limitations. First, it is a single‐arm trial without a control group, and it involves a relatively small patient cohort, which limits the ability to acquire definitive OS. Continued follow‐up and updated results are needed. Second, integrating artificial intelligence with multimodal data for efficacy prediction and explaining the underlying mechanism needs to be established. Third, due to difficulties in obtaining tissue samples from advanced breast cancer, only a limited number of patients can undergo genetic testing. Future studies should aim to expand the sample size or explore peripheral blood markers for further research.

In summary, this study suggests that the combination of pyrotinib and fulvestrant may provide an effective, well‐tolerated, and convenient treatment for HR‐positive/HER2‐positive metastatic breast cancer patients resistant to trastuzumab. This regimen could serve as a potential first‐line therapy, particularly for those with brain metastases, secondary trastuzumab resistance, or a history of adjuvant endocrine therapy. Furthermore, patients with low TMB and a *ZNF217* mutation may experience greater therapeutic benefit from this combination.

## 4 MATERIALS AND METHODS

### 4.1 Study design and participants

This prospective, multicenter, single‐arm phase II study (ClinicalTrials.gov identifier: NCT04034589) was conducted across five hospitals in China, including Sun Yat‐sen Memorial Hospital of Sun Yat‐sen University, Shenshan Medical Center of Memorial Hospital of Sun Yat‐sen University, The Affiliated Panyu Central Hospital of Guangzhou Medical University, Guangzhou Women and Children Medical Center, and The First People's Hospital of Zhaoqing. The study aimed to assess the effectiveness of combining pyrotinib with fulvestrant in patients with HR‐positive/HER2‐positive metastatic breast cancer following disease progression after trastuzumab treatment.

Eligible patients had HR‐positive/HER2‐positive metastatic breast cancer, with previous progression on trastuzumab. Primary resistance to trastuzumab refers to recurrence or metastasis that arises during adjuvant therapy or within one year of completing treatment, as well as disease progression within three months of initiating trastuzumab for advanced disease. In contrast, secondary resistance is defined by recurrence or metastasis under similar conditions, but with disease progression occurring after an initial positive response in advanced therapy. HER2 positivity was defined as an immunohistochemistry staining intensity of 3+ or *HER2* gene amplification determined by fluorescence in situ hybridization. HR positivity was defined as estrogen ER and/or PR immunohistochemical staining in more than 10% of tumor cells.

The main inclusion criteria were as follows: (1) Women with Eastern Cooperative Oncology Group performance status of 0 or 1. (2) Adequate organ function. (3) No previous systemic treatment for metastatic disease, except trastuzumab‐based first‐line therapy. Combined with ovarian function suppression (including bilateral oophorectomy and GnRHa) for premenopausal patients was allowed for study inclusion. Conversely, the main exclusion criteria were as follows: (1) Patients with active central nervous system metastases. (2) Patients who had previously received more than one line of endocrine therapy, chemotherapy, or targeted therapy for metastatic disease. (3) Female patients who were pregnant or breastfeeding, as well as fertile female patients with a positive baseline pregnancy test.

The study followed the Declaration of Helsinki and was approved by the ethics committee of Sun Yat‐sen Memorial Hospital in Guangzhou, China, along with those of all other participating institutions. All patients provided written informed consent.

### 4.2 Treatment procedures

All treatments were given in 28‐day cycles. Pyrotinib (provided by Hengrui company) 400 mg was taken orally once daily. Fulvestrant was administered as a 500 mg intramuscular injection on days 1 and 15 of cycle 1, followed by a 500 mg intramuscular injection once on day 1 of each subsequent monthly cycle. All participants continued study treatment until disease progression or relapse, unacceptable toxicity, an intercurrent illness that prevented further therapy, withdrawal of consent, or death, whichever occurred first. Tumor assessments were conducted every 12 weeks (±7 days) according to RECIST 1.1 criteria. Adverse events were assessed using version 4.0 of the National Cancer Institute's Common Terminology Criteria.

### 4.3 Outcomes

The primary endpoint was PFS, measured from randomization to the first confirmed progression or death from any cause, whichever occurred first. Secondary endpoints included the ORR, defined as the proportion of participants with confirmed complete or partial responses; DCR, encompassing objective responses or stable disease for at least 24 weeks; OS, calculated from randomization to death, with censoring at the last recorded date alive; and the safety profile, which evaluated the incidence and severity of adverse events (AEs). The exploratory study objective was the correlation of biomarkers with the efficacy and safety of pyrotinib and fulvestrant.

### 4.4 DNA extraction, library preparation, and sequencing

In this study, genomic DNA was extracted from 5‐µm tissue sections and white blood cells using QIAamp and DNeasy kits (Qiagen) followed by purification with the Promega Maxwell 16 Tissue LEV DNA kit. DNA quality and quantity were assessed with a NanoDrop 2000 (Thermo Fisher) and Qubit 2.0 (Life Technologies). Tumor DNA was fragmented into 300–350 bp using the Covaris M220, and sequencing libraries were prepared with the KAPA Hyper Prep Kit (KAPA Biosystems) and NEBNext kits (E6040S, NEB). Libraries were indexed, pooled, and enriched for 425 genes and 12,000 SNPs using xGen Lockdown Probes (IDT) and sized on a LabChip GX (Caliper), then quantified and sequenced on the HiSeq 4000 platform (Illumina).

Quality control of the FASTQ files was performed using Trimmomatic. The reads were aligned to the hg19 reference genome (Human Genome version 19) with Burrows‐Wheeler Aligner (BWA‐mem, v0.7.12). PCR duplicate reads were removed, and sequence metrics were collected using Picard (v1.47) and Samtools (v0.1.12a). Germline mutations in control blood samples were identified with GATK v3.4.0. Somatic mutations were detected using VarScan2 (minimum quality score = 15, somatic *p* = 0.1). Annotation was carried out using ANNOVAR with the hg19 reference genome and the 2014 standard databases. Genomic fusions were identified with FACTERA, while copy number variations were detected using ADTEx14 by comparing normal and tumor samples.

### 4.5 Statistical analyses

Considering the results of previous studies,^35^, ^36^ the PFS of “pyrotinib combined with fulvestrant” was 8.5 months and the precision of the confidence interval was 2.3 months, considering that the median survival time of 95% of the population was greater than 6.2 months. The percentage of study subject deletions was 50%. Using the PASS software, adopting the one‐sided test, the sample size was calculated for 46 individuals, which indicates 23 events (disease progression) will allow for the analysis of the main study endpoint of PFS.

Cumulative survival probabilities were estimated using the Kaplan–Meier method and compared via the log‐rank test, stratified by randomization strata. Hazard ratios with 95% CIs were derived from the stratified Cox proportional hazards model. The proportional hazards assumption was tested using Schoenfeld residuals. Prespecified exploratory subgroup analyses were performed based on prognostic factors such as age, HER2 status, ER status, PR status, treatment lines, and the presence of visceral, brain, or bone metastases, as well as prior adjuvant endocrine therapy, trastuzumab therapy, and trastuzumab resistance. The consistency of treatment effects across subgroups was assessed using an unadjusted Cox model. Acute toxicity rates were compared with two‐sample proportion tests. Efficacy and safety analyses included all patients who received the specified treatment. Missing data were not statistically imputed. All tests were two‐sided, with *p* < 0.05 indicating significance. Analyses were conducted using R v4.3.3 with data collected up to August 15, 2024.

## AUTHOR CONTRIBUTIONS

Ying Wang, Herui Yao, Kang Zhang, Zhongyu Yuan, and Jianli Zhao contributed to supervision, conceptualization, and project administration. Jianli Zhao, Yunfang Yu, Wei Ren, Linxiaoxiao Ding, and Yongjian Chen wrote and revised the manuscript. Jianli Zhao, Yunfang Yu, Wei Ren, Linxiaoxiao Ding, Yongjian Chen, and Peng Yuan contributed to data curation. Jian Yue, Yaping Yang, Guorong Zou, Tao Chen, Jie Chai, Li Zhang, Wenjing Wu, Yinduo Zeng, Xiujuan Gui, Yangyang Cai, and Simin Luo contributed to data curation and data analysis. All authors have read and approved the final manuscript.

## CONFLICT OF INTEREST STATEMENT

Kang Zhang, an editorial board member of Medcomm, did not participate in the review or decision‐making for this manuscript. The remaining authors declare no conflict of interest.

## ETHICS APPROVAL

The study adhered to the principles outlined in the Declaration of Helsinki and received approval from the ethics committee of Sun Yat‐sen Memorial Hospital, Sun Yat‐sen University (approval number: 2019‐KY‐049).

## Supporting information



Supporting information

## Data Availability

Data on patients that were used to support the findings of this study may be released upon application to the corresponding author.
