# Supplementary material for: Combined pyrotinib and fulvestrant for hormone receptor‐positive and HER2‐positive metastatic breast cancer: A multicenter, single‐arm, phase II trial
Source: MedComm (2020). 2024 Dec 20;6(1):e70031. doi: 10.1002/mco2.70031 (PMC11661908; doi:10.1002/mco2.70031)
Supplement: Supplementary file 1 — Supporting information [file MCO2-6-e70031-s001.docx]

**Data Supplement Content**

**Fig. S1 Kaplan-Meier curves for progression-free survival by the subcategory of bone metastases only.**

**Fig. S2 Confirmed objective response rate across subgroups.**

**Fig. S3 Oncoprint of individual gene mutations.**

**Table S1 Subgroup analysis of progression-free survival.**

**Table S2 Subgroup analysis of overall survival.**

**Fig. S1 Kaplan-Meier curves for progression-free survival by the subcategory of bone metastases.**

*P* values were calculated using the unadjusted log-rank test, and hazard ratios were calculated by a univariate Cox regression analysis. HR, hazard ratio.

**Fig. S2 Confirmed objective response rate across subgroups.**

**
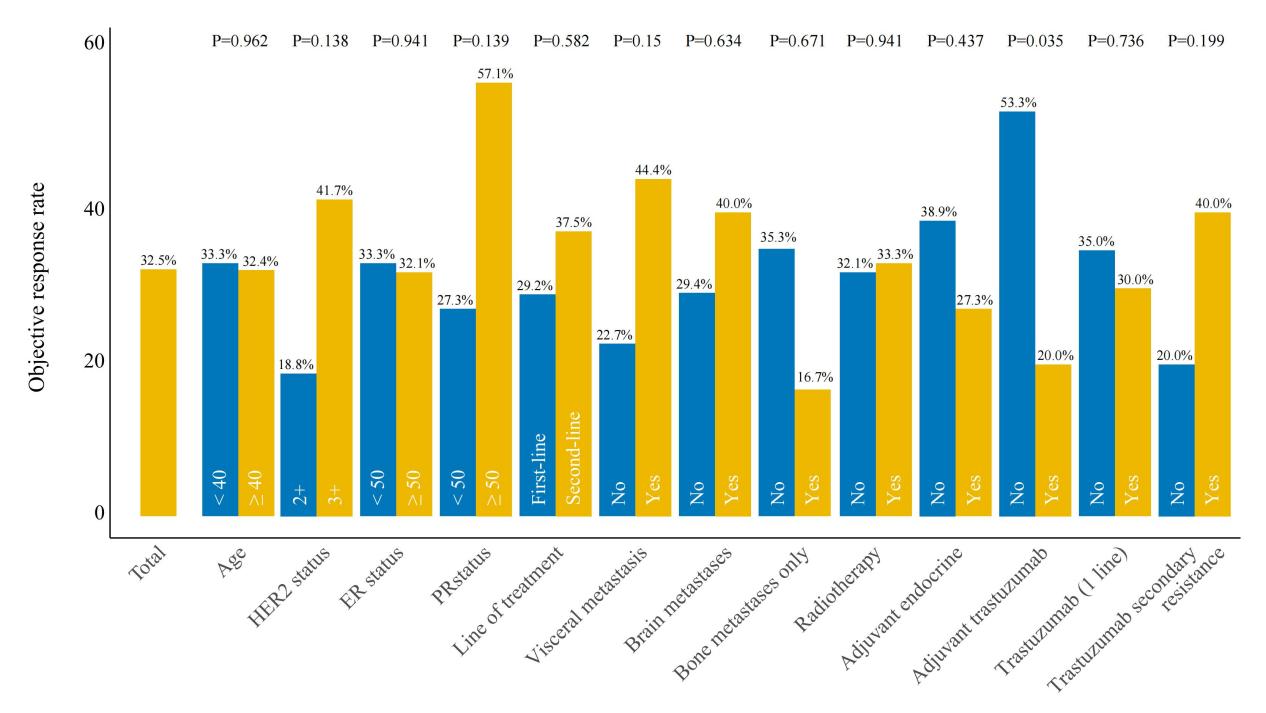
**

Response was determined by investigator assessment according to RECIST 1.1. ER, estrogen receptor; HER2, human epidermal growth factor receptor 2; PR, progesterone receptor.

**Fig. S3** **Oncoprint of individual gene mutations.**

**
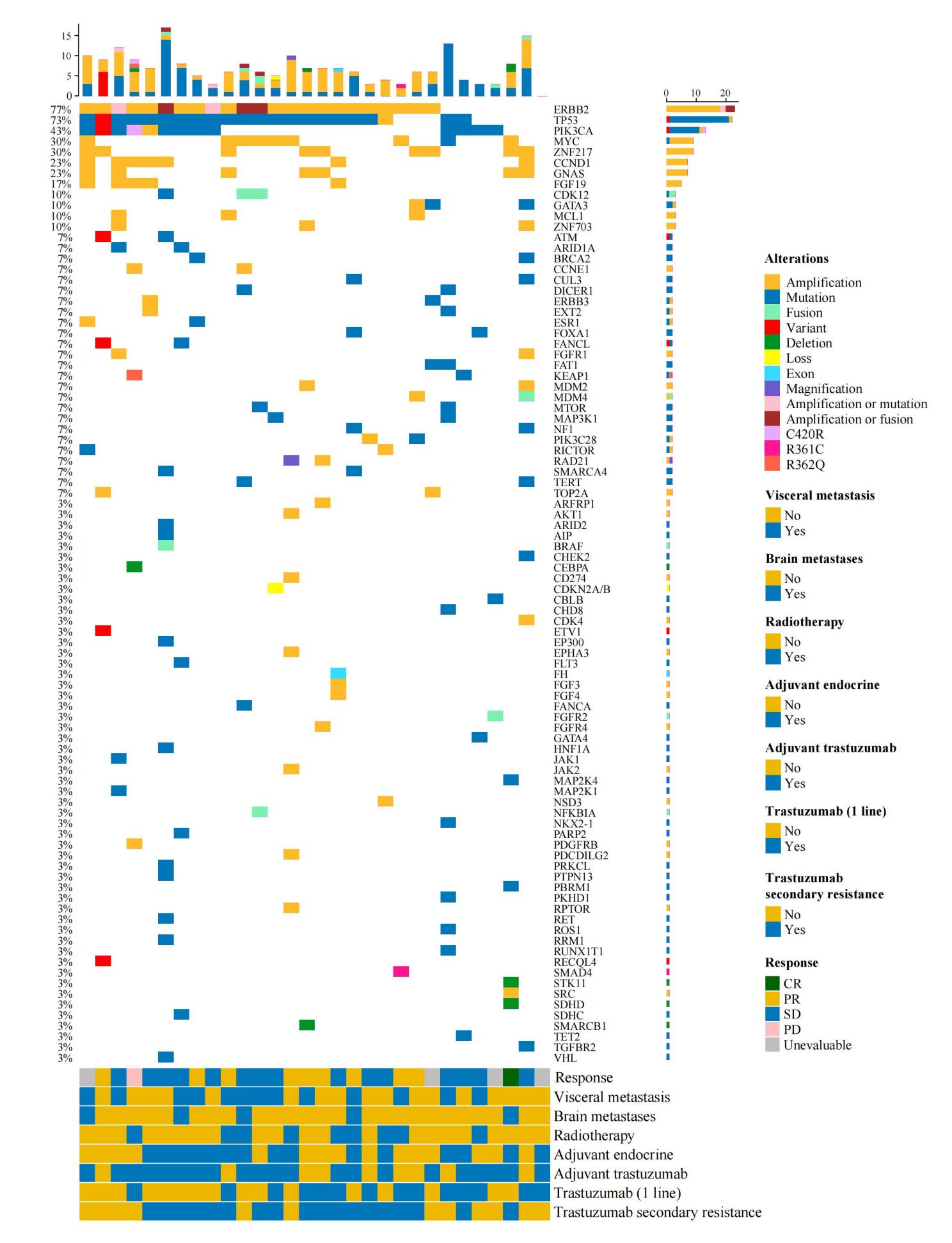
**

Unevaluable indicates bone metastases only were not measurable. CR, complete remission; PR, partial response; SD, stable disease; PD, progression disease.

**Table S1 Subgroup analysis of progression-free survival.**

| **Variable** | **Group** | **Median survival** | **HR (95%CI)** | ***P* value** |
| --- | --- | --- | --- | --- |
| **Age** | <40 | 17.67 (14.93 - NA) | 0.72 (0.27 - 1.91) | 0.507 |
|  | ≥40 | 19.07 (10.53 - NA) |  |  |
| **HER2 status** | 2+ | 16.73 (9.27 - NA) | 0.65 (0.31 - 1.36) | 0.250 |
|  | 3+ | 19.70 (11.93 - NA) |  |  |
| **ER status** | <50 | 18.17 (10.47 - NA) | 1.08 (0.49 - 2.38) | 0.851 |
|  | ≥50 | 17.67 (11.93 - NA) |  |  |
| **PR status** | <50 | 18.17 (10.60 - 31.07) | 1.10 (0.45 - 2.73) | 0.829 |
|  | ≥50 | 17.20 (9.57 - NA) |  |  |
| **Lines of treatment** | First | 19.53 (10.60 - NA) | 1.47 (0.71 - 3.07) | 0.302 |
|  | Second | 14.93 (10.47 - NA) |  |  |
| **Visceral metastasis** | No | 18.17 (10.53 - NA) | 0.85 (0.41 - 1.77) | 0.663 |
|  | Yes | 17.20 (10.60 - NA) |  |  |
| **Brain metastases** | No | 18.17 (10.47 - 41.73) | 0.96 (0.36 - 2.53) | 0.931 |
|  | Yes | 18.37 (16.73 - NA) |  |  |
| **Bone metastases only** | No | 17.20 (10.60 - 19.70) | 0.18 (0.04 - 0.79) | 0.011 |
|  | Yes | NA |  |  |
| **Radiotherapy** | No | 17.67 (10.60 - NA) | 0.70 (0.31 - 1.60) | 0.396 |
|  | Yes | 22.37 (10.53 - NA) |  |  |
| **Adjuvant endocrine** | No | 11.93 (9.27 - NA) | 0.50 (0.24 - 1.05) | 0.069 |
|  | Yes | 19.70 (17.67 - NA) |  |  |
| **Adjuvant trastuzumab** | No | 17.20 (11.93 - NA) | 0.96 (0.44 - 2.08) | 0.913 |
|  | Yes | 19.07 (10.53 - NA) |  |  |
| **Trastuzumab (1 line)** | No | 19.53 (16.73-NA) | 1.20 (0.57 - 2.50) | 0.631 |
|  | Yes | 14.93 (10.47-NA) |  |  |
| **Trastuzumab secondary resistance** | No | 14.93 (8.13-NA) | 0.59 (0.28 - 1.25) | 0.165 |
|  | Yes | 19.53 (17.20-NA) |  |  |

Abbreviations: CI, confidence interval; ER, estrogen receptor; HR, hazard ratio; HER2, human epidermal growth factor receptor 2; PR, progesterone receptor.

**Table S2 Subgroup analysis of overall survival.**

| **Variable** | **HR (95%CI)** | ***P* value** |
| --- | --- | --- |
| **Age** (≥40/<40) | 1.71 (0.22 - 13.51) | 0.610 |
| **HER2 status** (3+/2+) | 0.41 (0.12 - 1.46) | 0.170 |
| **ER status** (≥50/<50) | 0.94 (0.24 - 3.64) | 0.927 |
| **PR status** (≥50/<50) | 1.87 (0.48 - 7.25) | 0.366 |
| **Lines of treatment**  (Second-line/First-line) | 1.51 (0.44 - 5.24) | 0.514 |
| **Visceral metastasis** (Yes/No) | 1.32 (0.38 - 4.55) | 0.665 |
| **Brain metastases** (Yes/No) | 1.48 (0.31 - 6.99) | 0.621 |
| **Bone metastases only** (Yes/No) | 0.43 (0.05 - 3.42) | 0.413 |
| **Radiotherapy** (Yes/No) | 1.52 (0.43 - 5.44) | 0.515 |
| **Adjuvant endocrine** (Yes/No) | 0.17 (0.04 - 0.81) | 0.026 |
| **Adjuvant trastuzumab** (Yes/No) | 0.77 (0.22 - 2.74) | 0.688 |
| **Trastuzumab (1 line)** (Yes/No) | 1.58 (0.45 - 5.62) | 0.478 |
| **Trastuzumab secondary**  **resistance** (Yes/No) | 0.06 (0.01 - 0.49) | 0.008 |

Abbreviations: CI, confidence interval; ER, estrogen receptor; HR, hazard ratio; HER2, human epidermal growth factor receptor 2; PR, progesterone receptor.
